# Odontogenic necrotizing fasciitis: a systematic review of the literature

**DOI:** 10.1186/s12901-018-0059-y

**Published:** 2018-08-15

**Authors:** Mitchell R. Gore

**Affiliations:** SUNY Upstate Department of Otolaryngology, Syracuse, NY USA

**Keywords:** Odontogenic necrotizing fasciitis, Infection, Hyperbaric oxygen

## Abstract

**Background:**

While odontogenic soft tissue infections of the head and neck are common, progression to necrotizing fasciitis is relatively rare. Necrotizing fasciitis is a potentially life-threatening and rapidly progressive soft tissue infection that can lead to significant skin and soft tissue loss, mediastinitis, vascular thrombosis or rupture, limb loss, organ failure, and death.

**Methods:**

A PubMed literature search was conducted for case reports and case series on odontogenic necrotizing fasciitis. Individual patient data was analyzed and compiled and demographic, treatment, microbiology, and mortality data were extracted. Fisher’s exact test was used to examine the relationship between death from odontogenic necrotizing fasciitis and diabetes mellitus (DM) and human immunodeficiency virus (HIV) positivity.

**Results:**

A total of 58 studies totaling 164 patients were identified. Thirty-three patients had DM and 3 were HIV +. All patients underwent aggressive surgical debridement and treatment with IV antibiotics. Twenty patients were also treated with hyperbaric oxygen. There were 16 deaths reported, for a mortality rate of 9.8%. The mortality rate among patients with DM was 30.3 and 0% among HIV positive patients. There was a statistically significant increase in the mortality rate in DM patients with odontogenic necrotizing fasciitis (*p* = 0.0001, odds ratio for death 9.1).

**Conclusions:**

Necrotizing fasciitis arising from odontogenic infection is a rapidly progressive and life-threatening illness. Prompt recognition of the infection, aggressive and often serial surgical debridement, and aggressive broad-spectrum antibiotics are necessary to prevent serious morbidity and mortality. Patients with diabetes mellitus are at a significantly increased risk of death from odontogenic necrotizing fasciitis.

## Background

Necrotizing fasciitis is a potentially fatal soft tissue infection, characterized by extensive tissue necrosis and gas formation in the subcutaneous tissue, fascia, and deep tissues [1, 2]. It is most commonly bacterial in origin but sporadic cases caused by or associated with herpes zoster or herpes simplex have been reported [3–5]. In the head and neck region the etiology is most commonly odontogenic. Necrotizing fasciitis can spread rapidly, infiltrating tissue planes and leading to rapid progression with vascular compromise, organ failure, and mediastinitis or limb loss. The infection may be caused by myriad bacteria ranging from more common Streptococcal or Staphylococcal species to less common species such as Fusobacterium or Acinetobacter, and infections are often polymicrobial [1, 2]. The muscular, subdermal, and cutaneous vasculature often become compromised, leading to necrosis of muscle, skin, and loss of larger vessels. Underlying immune compromise such as diabetes mellitus (DM) or human immunodeficiency virus (HIV) infection may predispose patients to necrotizing fasciitis or may increase the risks or morbidity or mortality of necrotizing fasciitis [1, 2]. Patients with odontogenic necrotizing fasciitis often appear acutely ill and may have a history of recent dental procedures or dental or maxillofacial trauma, or long-standing dental neglect. Patients may present with fever, tachycardia, dehydration, hypotension, and skin and soft tissue may appear cyanotic, mottled, cellulitic, tense, or overtly necrotic. Management depends upon prompt diagnosis with aggressive surgical debridement and serial debridement of any ongoing necrosis, aggressive intravenous (IV) antibiotic therapy, and hemodynamic and airway support when needed. While odontogenic infections are commonplace, the progression to life-threatening necrotizing fasciitis is relatively rare, and thus may not be recognized by emergency room, medical, or surgical practitioners until the disease has progressed significantly. To our knowledge, no recent study has examined the aggregate literature on odontogenic necrotizing fasciitis and systematically reviewed its etiologic factors, bacteriology, treatment, and comorbidities such as HIV or diabetes mellitus that may impact survival. For this reason, in this study we sought to review the available literature on necrotizing fasciitis of odontogenic origin, and to examine patient demographics, the causative organisms, antimicrobials used, and to analyze the effect diabetes mellitus and HIV positivity have on mortality.

## Methods

**Fig. 1:**
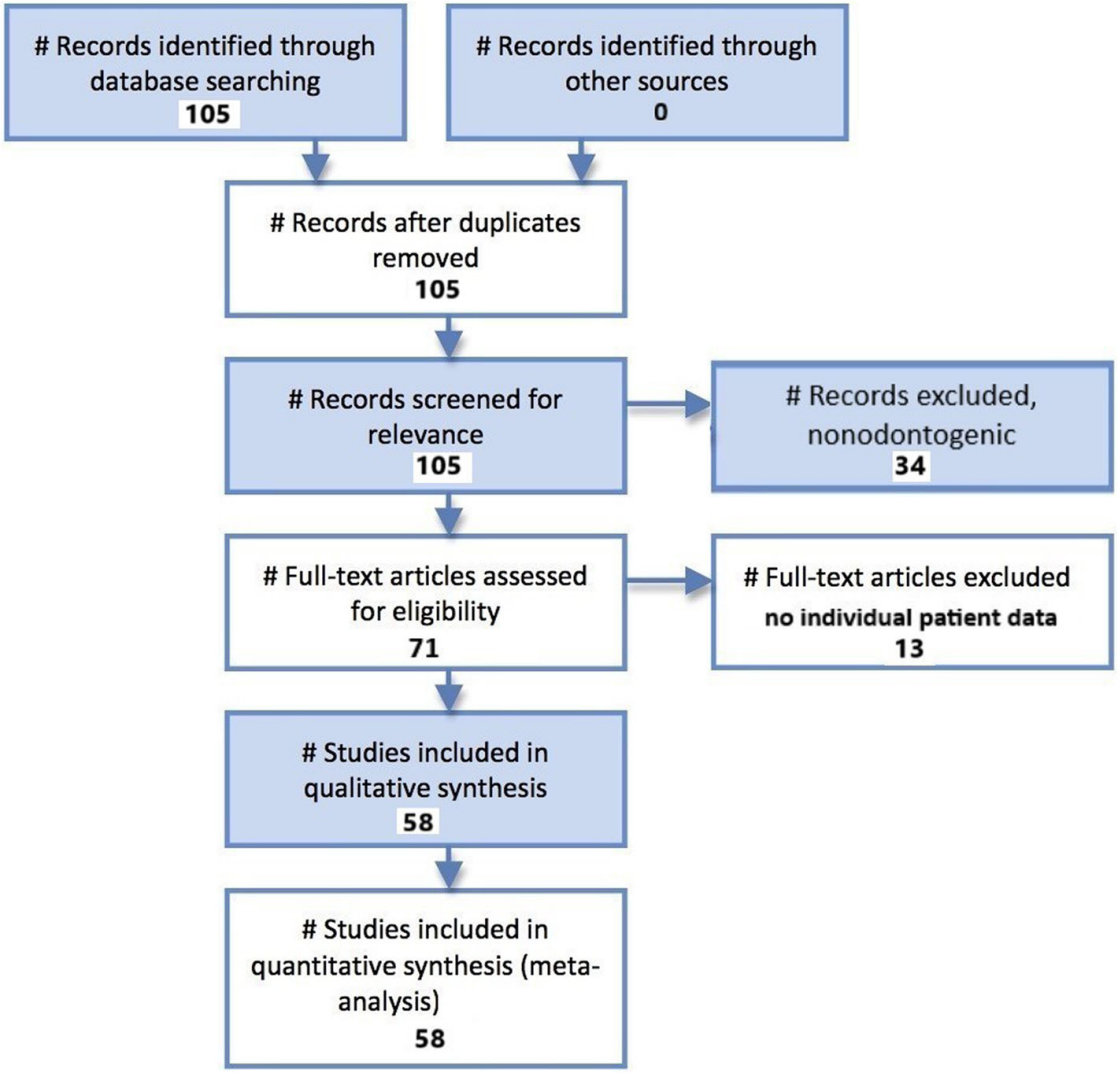
PRISMA flow diagram for odontogenic necrotizing fasciitis study selection

A Pubmed literature search was conducted using the search terms “odontogenic necrotizing fasciitis”. This literature review and meta-analysis was carried out and reported using the Preferred Reporting Items for Systematic Reviews and Meta-Analysis (PRISMA) guidelines for the reporting of observational studies [6]. Figure 1 illustrates the PRISMA flow diagram for study selection. For the period 1987–2018 105 total papers were identified. After excluding duplicate studies, nonodontogenic cases, review articles, and studies without analyzable individual patient data, a total of 58 studies reporting 164 total patients were identified [1, 7–63]. Case reports, case series, and cohort studies containing individual analyzable patient data on patients of any age with a pathologic diagnosis of odontogenic necrotizing fasciitis were included in the systematic review.

### Statistical analysis

Statistical analyses were performed with XLSTAT Biomed (Addinsoft, New York, NY, USA/Paris, France). Overall patient demographics, organisms isolated from culture, treatment type (surgical, antibiotic type, and use of hyperbaric oxygen) and mortality outcomes were compiled via standard summary statistical methods. Fisher’s exact test was used to compare non-DM and DM and HIV negative and HIV positive groups and to calculate the two-sided *P* values. P values less than 0.05 were considered statistically significant.

## Results

A total of 164 patients with odontogenic necrotizing fasciitis were identified with analyzable individual patient data. Sixty-five patients had data on sex reported, with 45 males and 20 females reported (2.3:1 M:F ratio). A total of 33 patients were reported to have DM (20.1%), while 3 were HIV+ (1.8%). The median and average ages were 45.0 and 46.3 years, respectively (standard deviation 15.9 years).

The most common microbes isolated from culture were mixed anaerobes (9.8%), unspecified streptococcus species (9.8%), and mixed microbiological flora (9.1%). Table 1 summarizes the culture data from the cohort. All patients were treated with surgical debridement along with IV antibiotics. The most common IV antibiotics reported were metronidazole (21.3%), clindamycin (13.4%), penicillin (11.0%), and ceftriaxone (7.9%). Table 2 summarizes the antibiotic data from the cohort. Twenty patients (12.2%) were treated with hyperbaric oxygen, and there were no reported deaths in the hyperbaric oxygen + surgery + IV antibiotics group. Hyperbaric oxygen treatment in addition to surgery + IV antibiotics was not associated with a statistically significant decrease in mortality vs. surgery + IV antibiotics alone (*p* = 0.2).

**Table 1:** Microbiological culture data from the odontogenic necrotizing fasciitis cohort

Microbe isolated	Number of patients (n)	% of patients
Mixed microbiological flora	15	9.10%
Prevotella buccae	1	0.60%
*Escherichia coli*	1	0.60%
*Staphylococcus aureus*	6	3.70%
Staphylococcus capitis	1	0.60%
alpha hemolytic streptococci	3	1.80%
Prevotella species unspecified	7	4.30%
Peptostreptococcus	7	4.30%
coagulase-negative Staphylococcus	7	4.30%
Corynebacterium	1	0.60%
Methicillin-resistant Staphylococcus aureus	2	1.20%
mixed anaerobes	16	9.80%
Gram-negative species	6	3.70%
*Streptococcus anginosus*	1	0.60%
Bacteroides species	1	0.60%
*Klebsiella pneumoniae*	3	1.80%
Streptococcus pyogenes	4	2.40%
Acinetobacter	1	0.60%
Fusobacterium	1	0.60%
*Staphylococcus epidermidis*	1	0.60%
no growth	1	0.60%
Streptococcus group C	1	0.60%
Streptococcus viridans	1	0.60%
Streptococcus species unspecified	16	9.80%
Diptheroids	1	0.60%
Streptococcus milleri	2	1.20%
Streptococcus sangius	1	0.60%
Streptococcus porphyromonas	1	0.60%

**Table 2:** Intravenous antibiotic usage data from the odontogenic necrotizing fasciitis cohort

Antibiotic used	Number of patients (n)	% of patients
ceftriaxone	13	7.90%
metronidazole	35	21.30%
ciprofloxacin	3	1.80%
clindamycin	22	13.40%
imipenem	1	0.60%
vancomycin	1	0.60%
penicillin	18	11.00%
Ampicillin + sulbactam	2	1.20%
cefotaxime	2	1.20%
gentamicin	5	3.00%
amoxicillin-clavulanate	2	1.20%
amikacin	1	0.60%
amoxicillin	1	0.60%
ceftazidime	5	3.00%
cefuroxime	1	0.60%

There were 16 total deaths reported for a mortality rate of 9.8%. Of the 33 patients with diabetes mellitus there were 10 deaths (30.3%). Of the 131 non-DM patients there were 6 deaths (4.6%). Of the 3 patients who were HIV+ there were no deaths. The odds ratio for death for odontogenic necrotizing fasciitis patients with DM vs. those without DM was 9.1 (95% confidence interval (CI) =3.0 to 27.4, *p* = 0.0001). The number needed to treat/number needed to harm (NNT/NNH) was 4.0. The odds ratio for death for odontogenic necrotizing fasciitis patients with HIV vs. HIV- patients was 1.0 (95% CI = 0.05 to 19.7, *p* = 1.0, NNT/NNH = 8.0). Table 3 summarizes the odds ratio analysis for DM and HIV in patients with odontogenic necrotizing fasciitis.

**Table 3:** Odds ratio analysis for mortality risk with DM and HIV in patients with odontogenic necrotizing fasciitis

Diabetes mellitus	
Odds ratio for death	9.1
95% CI of odds ratio:	3.0 to 27.4
Significance level	*P* = 0.0001
NNT/NNH	3.9
HIV	
Odds ratio for death	0.98
95% CI of odds ratio:	0.05 to 19.7
Significance level	*P* = 0.99
NNT/NNH	8

## Discussion

Necrotizing fasciitis is an aggressive soft tissue infection that can be polymicrobial or due to a single organism [1, 2, 11, 64]. Multiple organisms as well as mixed infections have been reported to cause necrotizing fasciitis of odontogenic origin. In the present study there were 30 different distinct microbiological results isolated from cultures taken from patients with odontogenic necrotizing fasciitis. The microbes isolated ranged from multiple species of Staphylococcus and Streptococcus to mixed anaerobic species and less common bacteria such as Prevotella and Fusobacterium.

Odontogenic necrotizing fasciitis is often characterized by rapidly progressive bacterial infection along multiple fascial tissue planes, leading to vascular compromise, thrombosis, or rupture, along with necrosis of adipose, integumentary, muscular, and subcutaneous and cutaneous tissues. Preexisting immunosuppressive conditions such as diabetes mellitus may predispose patients to odontogenic necrotizing fasciitis, and may increase the mortality risk [1, 2, 11, 64]. In the present study approximately 20% of patients were reported to have DM, and these patients were 9 times more likely to die from their odontogenic necrotizing fasciitis than non-diabetic patients (*p* = 0.0001). This highlights the need to identify co-morbidities such as DM and treat them appropriately while the patient is concomitantly being treated for odontogenic necrotizing fasciitis. In contrast to DM, only 1.8% of patients in the present study had HIV. These patients did not have an increased risk of death from odontogenic necrotizing fasciitis vs. HIV negative patients (OR = 1.0, *p* = 1.0). While most cases of head and neck necrotizing fasciitis are odontogenic in origin, idiopathic or pharyngeal etiologies are possible.

Patients with odontogenic necrotizing fasciitis should be treated aggressively with surgical debridement of necrotic tissue and close monitoring with serial debridement and/or frequent dressing changes as indicated. Broad-spectrum IV antibiotics targeting the most common organisms are also vital. In the present study all patients were treated with surgical debridement and IV antibiotics. The most common antibiotics were metronidazole, clindamycin, penicillin, and ceftriaxone but antimicrobial treatment may need to be adjusted once culture results are available in a given case. Debridement of necrotic tissue until viable tissue that bleeds is reached is typically recommended, although care must be taken in necrotizing fasciitis in close proximity to the great vessels, mediastinum, or lungs. The 9.8% overall mortality rate in this study highlights the importance of aggressive treatment with surgery and IV antibiotics. In this study 12.2% of patients were also treated with hyperbaric oxygen, which may be a useful adjunct in refractory cases and when hyperbaric oxygen is readily available. Critical care team involvement is often necessary, and airway management and management of hypotension, hypovolemia, and malnutrition may be necessary in patients with odontogenic necrotizing fasciitis.

Prompt recognition of odontogenic infections that have progressed to necrotizing fasciitis is key. While typical odontogenic infections such as cellulitis and periapical or cervical abscess may present with common or nonspecific symptoms such as swelling, pain, and trismus patients with dusky, tense, insensate, crepitant, or mottled skin, or evidence of involvement of multiple fascial planes and tissue compartments or evidence of gas formation on CT or MRI imaging may have concomitant necrotizing fasciitis needing more aggressive treatment. Survivors of odontogenic necrotizing fasciitis may also have extensive skin and soft tissue loss that may necessitate weeks to months of dressing changes or secondary reconstructive procedures such as skin grafts. Additionally, chronically poor dentition or carious teeth may need to be addressed by dental or oral surgery consultants to prevent recurrence.

The clinical implications of this study are several. The study demonstrates that patients with DM are at greater risk for morbidity and mortality in the setting of odontogenic necrotizing fasciitis. This highlights the need for recognition of DM as a comorbidity using serum glucose testing and hemoglobin A1C testing to assess known and newly diagnosed diabetics. Additionally, diabetics should have tight glucose control during their treatment for odontogenic necrotizing fasciitis, as this may impact the efficacy of their treatment and may affect the length of treatment. The study also demonstrates that a myriad of microorganisms can be causative agents in odontogenic necrotizing fasciitis, making cultures and sensitivities along with broad spectrum antibiotics transitioning to culture-directed antibiotics vital. The study also reiterates the need for aggressive surgical treatment and debridement to remove diseased, necrotic tissue. Extensive reconstructive procedures may be necessary once the necrotizing fasciitis is resolved, but surgical debridement to prevent mortality is of paramount importance. Additionally, the study suggests that adjunctive measures such as hyperbaric oxygen may be beneficial in cases where the patient is stable enough to undergo these procedures, and they are readily available to the treatment team.

The retrospective data in the study makes recall and selection bias a possibility. Nevertheless, the relatively large cohort (164 patients) for this relatively rare disease, combined with the highly significant mortality increase for patients with DM and the consistent therapy applied across the cohort (all patients were treated with surgical debridement and IV antibiotics) supports the validity of the study and its conclusions. The rarity of odontogenic necrotizing fasciitis makes prospective randomized trials difficult to assemble and the often rapid mortality and morbidity might make such trials ethically unfeasible, so relatively large patient cohort, rigorous statistical analysis, and diverse patient base represented in the literature makes this retrospective study a useful addition to the body of scientific knowledge on odontogenic necrotizing fasciitis.

## Conclusions

Odontogenic necrotizing fasciitis is a relatively uncommon but life-threatening and rapidly progressive illness. The present study showed an overall mortality rate of 9.8%, with a significantly increased mortality rate of 30.3% in patients with diabetes mellitus. HIV infection did not appear to increase the risk of death from odontogenic necrotizing fasciitis in the present study. Management of odontogenic necrotizing fasciitis should involve aggressive and often serial surgical debridement, broad-spectrum IV antibiotics, resuscitation as indicated and management of concomitant systemic conditions such as diabetes. In patients with resultant skin or soft tissue loss physical rehabilitation and reconstructive procedures may also be needed. Hyperbaric oxygen may be a useful adjunctive treatment to surgery and IV antibiotics.

## Abbreviations

CI: Confidence interval
DM: Diabetes mellitus
HIV: Human immunodeficiency virus
IV: Intravenous
NNT/NNH: Number needed to treat/number needed to harm
PRISMA: Preferred Reporting Items for Systematic Reviews and Meta-Analyses
